# The Potential of Toll-Like Receptors to Modulate Avian Immune System: Exploring the Effects of Genetic Variants and Phytonutrients

**DOI:** 10.3389/fgene.2021.671235

**Published:** 2021-08-26

**Authors:** Muhammad Saif-ur Rehman, Saif ur Rehman, Wasim Yousaf, Faiz-ul Hassan, Waqas Ahmad, Qingyou Liu, Hongping Pan

**Affiliations:** ^1^State Key Laboratory for Conservation and Utilization of Subtropical Agro-Bioresources, Guangxi University, Nanning, China; ^2^Faculty of Animal Husbandry, Institute of Animal and Dairy Sciences, University of Agriculture, Faisalabad, Pakistan; ^3^Department of Clinical Sciences, University College of Veterinary and Animal Sciences, Narowal, Pakistan

**Keywords:** chicken, toll-like receptors, gene expression, nutrigenomic, polymorphism, immunity

## Abstract

Toll-like receptors (TLRs) are pathogen recognition receptors, and primitive sources of innate immune response that also play key roles in the defense mechanism against infectious diseases. About 10 different TLRs have been discovered in chicken that recognize ligands and participate in TLR signaling pathways. Research findings related to TLRs revealed new approaches to understand the fundamental mechanisms of the immune system, patterns of resistance against diseases, and the role of TLR-specific pathways in nutrient metabolism in chicken. In particular, the uses of specific feed ingredients encourage molecular biologists to exploit the relationship between nutrients (including different phytochemicals) and TLRs to modulate immunity in chicken. Phytonutrients and prebiotics are noteworthy dietary components to promote immunity and the production of disease-resistant chicken. Supplementations of yeast-derived products have also been extensively studied to enhance innate immunity during the last decade. Such interventions pave the way to explore nutrigenomic approaches for healthy and profitable chicken production. Additionally, single-nucleotide polymorphisms in TLRs have shown potential association with few disease outbreaks in chickens. This review aimed to provide insights into the key roles of TLRs in the immune response and discuss the potential applications of these TLRs for genomic and nutritional interventions to improve health, and resistance against different fatal diseases in chicken.

## Introduction

Innate and adaptive immunity have been considered as largely separate through complementary mechanisms of defense against microbes (Tipping, 2006). Both immune systems distinguish foreign organisms as non-self and trigger the corresponding defence action. Specifically, antigen receptors on lymphocytes are key feature of adaptive immunity, while innate immunity relies on antigen presenting cells (APCs) and phagocytic cells including dendritic cells (DCs), granulocytes, and macrophages (Tate et al., 2010). Macrophages and DCs belong to the innate immune cells that are activated by microbial components, such as lipopolysaccharide (LPS) of Gram-negative bacteria (Tate et al., 2010). Macrophages perform functions, such as phagocytosis, production of chemokines and cytokine, secretion of antimicrobial peptides, and antigen presentation (Parihar et al., 2010).

Toll-like receptors (TLRs) are a group of pattern recognition receptors (PRRs) and are also the main components of innate immunity (Kawai and Akira, 2010). These receptors provide protection against a wide range of pathogens (Zhang and Liang, 2016). TLRs modulate signaling pathways in the host defence system to control the infection and repair damaged cells (Wang et al., 2016). Agonists/ligands are specialized structural motifs present on microbes that activate the macrophages upon binding with the corresponding TLRs. The binding of specific ligands to TLRs activates various adaptor proteins, transcriptional factors, and stimulates cytokine genes (Kawasaki and Kawai, 2014). These cytokines increase inflammatory responses and protect against various diseases (Bresnahan and Tanumihardjo, 2014). There are multiple factors that can regulate the functions of TLRs including genetic polymorphism and nutrients (Vidya et al., 2017; El-Zayat et al., 2019a).

Genetic associations among different TLRs can help scientists to study the genetic potential and prevention of infectious diseases in birds. To date, 10 TLRs genes have been characterized in chicken (Roach et al., 2005; Yilmaz et al., 2005; Iqbal et al., 2005b; Higgs et al., 2006). Variations in the sequence of TLRs change the recognition patterns of PAMPs and modify the host resistance against pathogenic infections (Ruan and Zheng, 2011; Ruan et al., 2015). TLRs polymorphism can play a crucial role as a genetic marker for the selective breed improvement programs in chicken. This literature review highlights the key roles of TLRs in the immune mechanisms, and further describes potential applications of these TLRs for genomic and nutritional interventions to improve health and resistance against different infectious diseases in chicken.

## Pattern Recognition Receptors

Phagocytic recognition of invading microbes activates inflammatory reactions against these pathogens *via* a set of PRRs including the scavenger receptors, TLRs, complement receptors, integrins, and members of the C-type lectin receptor family. These germline receptors are specialized structures that change over time to identify specific motifs on pathogens that are absent in higher-order eukaryotes, and these motifs control the invading pathogens (Aderem and Smith, 2004). TLRs are conserved transmembrane proteins of the PRRs family and categorized by the presence of an extracellular domain, which further contains LRR and a cytoplasmic domain (TIR domain; Akira et al., 2006).

**Figure 1:**
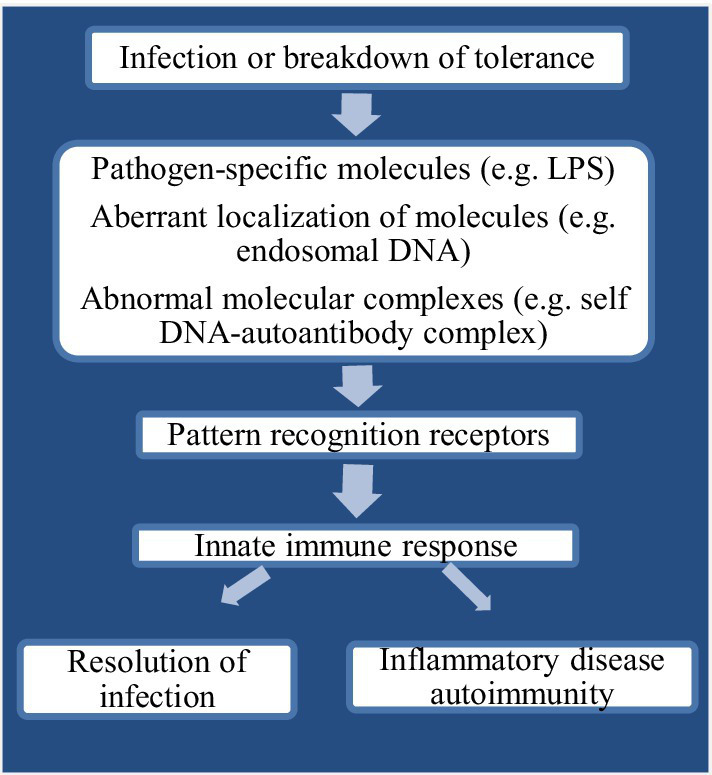
Flow chart diagram showing innate immune recognition through pattern recognition receptors (PRRs).

PAMPs have usually been found invariant which also help pathogens in survival and support to maintain divergent feature from “self” (Haunshi and Cheng, 2014). Recognition of “alert signals” in the host during aberrant localization or presence of inflammatory molecules or different cellular stress is mostly assisted by PRRs (Beg, 2002). Upon recognition by PAMPs, PRRs indicate the presence of infectious agents either by presentation at the cell surface or activate antimicrobial and proinflammatory responses through a signal to the host immune system (Figure 1). This mechanism further elicits several intracellular signaling pathways, such as kinases, transcription factors, and adaptor molecules (Akira and Takeda, 2004). The chicken TLRs (chTLRs) also recognize specific components of pathogens (agonist) as shown in Table 1.

**Table 1:** Major chTLRs receptors.

TLR	Ligands	Pathogen	Accession No.	References
TLR1A/TLR1 Type 1/TLR16	Lipoprotein	Bacteria	AB109401	Yilmaz et al., 2005
TLR1B/TLR1 Type 2	Lipoprotein	Mycoplasma	DQ518918	Yilmaz et al., 2005
TLR2A/TLR2 Type 1	Lipoprotein and peptidoglycans	Bacteria and fungus	NM_204278	Higgs et al., 2006
TLR2B/TLR2 Type 2	Lipoprotein and peptidoglycans	Bacteria and fungus	AB046533	Higgs et al., 2006
TLR3	dsRNA	Viruses	NM_001011691	Iqbal et al., 2005b; Roach et al., 2005; Higgs et al., 2006
TLR4	LPS	Bacteria	AY064697	Iqbal et al., 2005b; Roach et al., 2005; Higgs et al., 2006
TLR5	Flagellin	Bacteria	AJ626848	Iqbal et al., 2005b; Higgs et al., 2006
TLR7	ssRNA	Viruses	NM_001011688	Philbin et al., 2005; Roach et al., 2005; Higgs et al., 2006
TLR15	Lipoprotein	Yeast	NM_001037835	Higgs et al., 2006
TLR21	CpG ODN	Bacteria and viruses	NM_001030558	Roach et al., 2005

## Toll-Like Receptors

First TLR was identified in Drosophila (Chu and Mazmanian, 2013). Later on, the regulation of innate immunity was also observed in fungal infections (Plato et al., 2015). TLR is a primitive pathogen scrutiny system and widespread in animals and plants. At present, 13 receptors have been recognized in mammals, but TLR 10, 11, and 13 have exhibited specie specific gene expression (Leulier and Lemaitre, 2008). Many vertebrate contains 10 to 13 TLRs (Roach et al., 2005), e.g., humans carry TLR1-10 while TLR1-13 are present in mice. The chTLRs gene expression has demonstrated their conserved nature during evolution. At least 10 diverse TLRs have been recognized in chicken so far, but they express differently in contrast to mammals as shown in Table 1 (Brownlie and Allan, 2011). These chTLRs are originated from gene duplication, a phenomenon known as paralogs (Roach et al., 2005), and these paralogs are further classified into six major groups through phylogenetic analysis; TLR2 cluster (include TLRs 1, 2, 6, 10, and 14), TLR3, TLRs4, TLRs5, TLRs7/8/9, and lastly, TLR11 cluster that contains TLRs 11, 12, 13, 21, 22, and 23, respectively.

ChTLRs are slightly different from other vertebrates due to the presence of the pseudogene TLR8, chTLR1LA, chTLR1LB, chTLR15 and chTLR2, and the absence of TLR9 (Brownlie and Allan, 2011). Explicitly, orthologs to mammals have been characterized in chicken, such as for mTLR3, mTLR4, mTLR5, and mTLR7, whereas chicken lacks TLR8/9. However, mTLR1, mTLR6, and mTLR10 are exchanged with TLR1A and B, while TLR2A and TLR2B are replaced with TLR2 because of gene doubling. TLR15 and TLR21 are the two additional chTLRs. Avian species have distinct TLR15, which is stimulated after the invasion of bacterial proteases and virulent fungi (Iqbal et al., 2005a; Yilmaz et al., 2005; Temperley et al., 2008; de Zoete et al., 2010), while TLR21 is discrete from mTLR9 which has chief orthologs in amphibians and fish (Brownlie et al., 2009).

## Tlrs Mediated Immune Response

The primary function of TLRs during innate immune actions is similar in both birds and mammalian species, and APCs surface assists TLRs for their expression. TLRs identify PAMPs and establish signaling to the innate immune response by stimulating the reactive oxygen and nitrogen intermediates. APCs stimulate the upregulation of co-stimulatory molecules and pro-inflammatory cytokines (Takeda and Akira, 2005). APCs also play a role of junction between innate and adaptive immunity in pathway stimulated by TLRs. Although, peripheral tissues have immature DCs that are considered as affinity sites for microbial invasion (Mbongue et al., 2014), stimulation of TLRs results in the maturation of immature DCs that not only regulate major histocompatibility complex molecules and co-stimulatory molecules, but also boost lymphoid organs to stimulate T cells for antigen activation. Cellular activation is controlled by special receptors “chemokine” which are activated by DCs and upregulate TLRs (Cutler et al., 2001).

**Figure 2:**
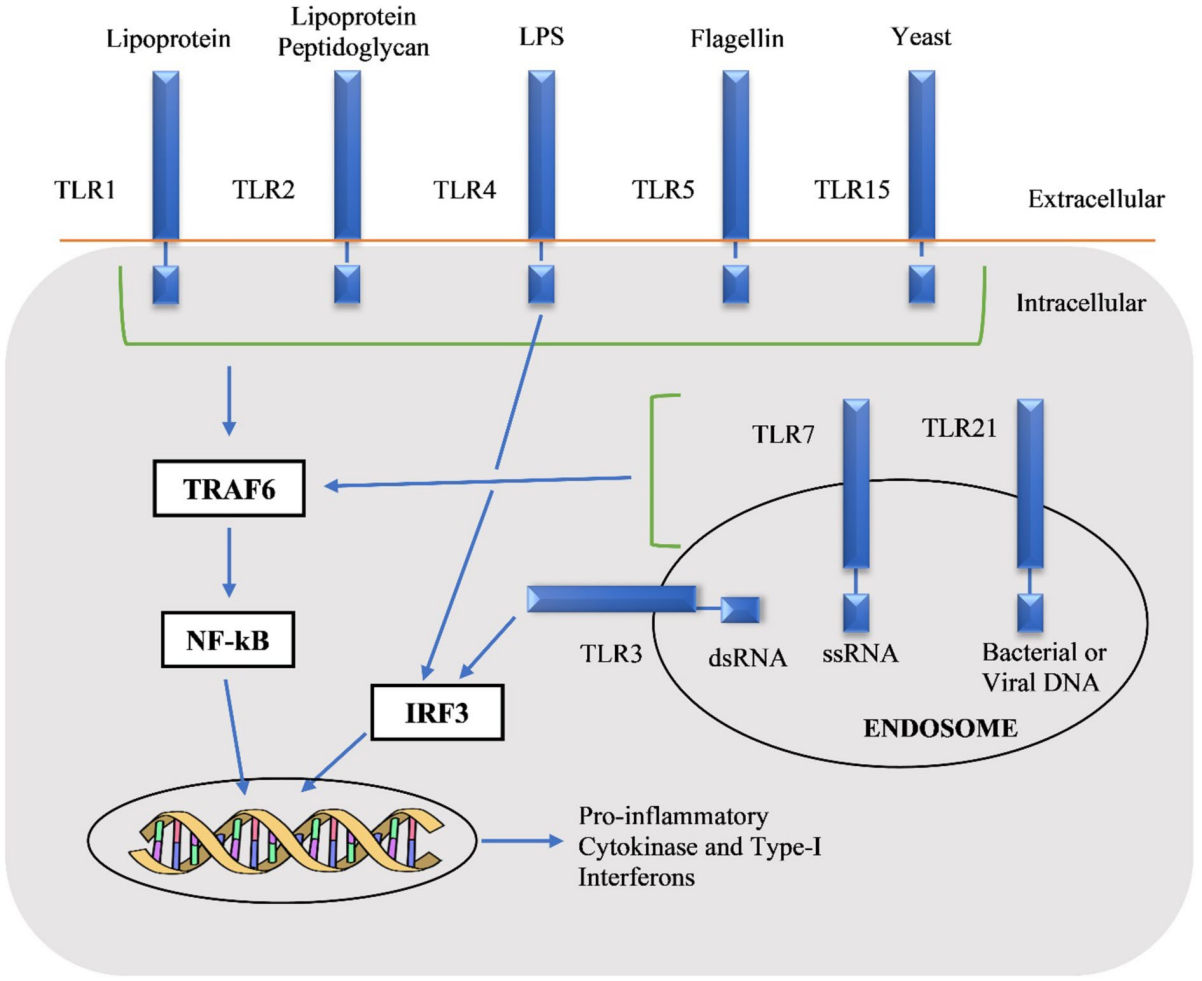
Pathways of chicken toll-like receptors (chTLRs) by recognition of their agonists (Gupta et al., 2014).

The chTLRs stimulate multiple pro-inflammatory cytokines through monocytes like interleukin macrophage inflammatory protein-1β, IL-6, IL-1β, and IL-8 and these cytokines have innate immune and inflammatory responses (He et al., 2011). The first step in TLR signaling is the myeloid differentiation protein 88 (MyD88)-dependent pathway which in return activates mitogen-activated protein kinase (MAPK), nuclear factor-kappa B (NF-κB), following the production of inflammatory chemokines and cytokines. The second pathway is MyD88-independent that stimulates various factors including interferon-inducible genes, type-1 interferons (IFN) as shown in Figure 2 (Takeda and Akira, 2005; Kawai and Akira, 2006). The expression of antimicrobial peptides is due to the TLR signaling pathway and these are the major molecules of innate immune systems. Taken together, these observations explain the potential role of TLRs to modulate innate and adaptive immunity, and this intrinsic property of TLRs can be used as adjuvants in vaccines (Birchler et al., 2001).

## Tlr Signaling in Chicken Diseases

The chTLRs expression has been studied against several pathogens and their association was found with disease resistance. The most important chTLRs are discussed below:

### Toll-Like Receptor2 (Type 1 and 2)

The chTLR2 was reported for the first time in 2001 and grouped into two types, TLR2 type 1 and type 2 located on chromosome 4; TLR1 is also classified in a similar manner (Fukui et al., 2001; Yilmaz et al., 2005), and chTLR2 type 1 and 2 are analogous to hTLR2, perhaps due to gene duplication (Plato et al., 2015). The expression pattern of chTLR2 revealed that it is only expressed in caecal tonsils, spleen, liver, bursa, B cells, CD8+ cells, and heterophils (Iqbal et al., 2005a; Kogut et al., 2008). A number of ligands bind to chTLR2 because of heterodimerization of chTLR2 homologs with TLR1 isoforms, and these isoformic properties of TLRs initiate activity to other agonists. Therefore, chTLR2/TLR1 heterodimerization upregulates the activity against synthetic tri-acylated lipopeptide in chicken, whereas chTLR2-1/TLR1-2 heterodimerization activates upon binding with peptidoglycans (Keestra et al., 2007; Higuchi et al., 2008). The TLR2 upregulates initial response of cytokine in host cells upon recognition of *M. pulmonis* (Love et al., 2010). ChTLR2 response was significantly highest in Hela 57A against *Campylobacter* spp. (*p*<0.05) when treated with lysed *Campylobacter* (de Zoete et al., 2010). In addition, TLR2 members were also associated with *Clostridium perfringens* and their expression was upregulated in the spleen. Moreover, the combined gene expression levels of TLR2.2 and TLR1.1 were also higher which also suggested the role of TLR1 in the regulation of innate host response (Lu et al., 2009).

### Toll-Like Receptor3

The chTLR3 is located on chromosome 4 (Yilmaz et al., 2005) and expressed in the thymus, duodenum, ileum, jejunum, colon, caecum, B cells, T cells, and heterophils (Iqbal et al., 2005a). In chicken, it binds with polyinosinic polycytidylic [Poly (I:C)] acid (He et al., 2007, 2012; Schwarz et al., 2007). The said acid is a double-stranded RNA (dsRNA) homolog that induces stimulation of IL-10, IFN-α, IFN-γ, and IFN-β similar to mammals. However, it produces a scanty amount of nitric oxide in monocytes and also decreases the activation of pro-inflammatory cytokines (He et al., 2012).

### Toll-Like Receptor4

ChTLR4 recognizes a major structural component of the cell wall of Gram-negative bacteria which is known as LPS and stimulates the immune system. Upon interaction with the extracellular proteins, LPS makes a complex of myeloid differentiation protein 2 (MD2), cluster of differentiation 14 (CD14), and TLR4 (Oshiumi et al., 2003). TLR4 and MD2 have 43 and 31% similarity with protein orthologs present in humans, respectively, and both were cloned in HeLa cells, and their expression profiles are noteworthy (Keestra et al., 2007). LPS binds with TLR4 to induce a cascade of signaling pathways following activation of NF-κB that results in pro-inflammatory chemokines and cytokines production (Fukui et al., 2001). TLR4 signaling also produces many chemokines, cytokines, IL-6, IL-8, and IL-1β upon invasion of viral and bacterial pathogens (Heggen et al., 2000; Kaiser et al., 2000; Laurent et al., 2001). ChTLR4 has a range of expression pattern, but its genetic expression level is higher in heterophils and macrophages (Leveque et al., 2003; Kogut et al., 2005; Iqbal et al., 2005a).

### Toll-Like Receptor5

ChTLR5 is activated by bacterial flagellin and plays a key function in host resistance to pathogenic bacteria (Hayashi et al., 2001; Wlasiuk et al., 2009). It is located on chromosome 3 and possesses almost 50% homology with hTLR5 at amino acid level (Iqbal et al., 2005a,b). Upregulated gene expression of chTLR5 has been observed in a few tissues that induces pro-inflammatory responses. Flagellin-based stimulation of Stromal and HD11 cells results in the production of IL1-β (Iqbal et al., 2005b). In heterophils, the expression levels of inflammatory chemokines and IL6 are increased upon binding with LPS/flagellin (Kogut et al., 2008). TLR5 expresses in HeLa cells and activates NF-κB when treated with purified flagellin or *Salmonella enterica serovar Enteritidis* (Keestra et al., 2008). ChTLR5-mediated molecular mechanisms may contribute to lung infection and cause acute respiratory syndrome (Zhang et al., 2012). However, few bacteria like *Campylobacter Jejuni* do not carry TLR5-binding sites that make chTLR5 sense them because of variation in flagellin (Paul et al., 2013).

### Toll-Like Receptor7

ChTLR7 is alternatively spliced that owes 63% similarity with chTLR7 at amino acid level and expressed in the forms of three different transcripts to produce two types of proteins (Philbin et al., 2005). ChTLR7 is expressable in a number of cells/tissues likewise other TLRs (Iqbal et al., 2005a; Philbin et al., 2005). Single-stranded RNA has been identified as a ligand for TLR7 which is controlled by a gene similar to TLR8 (Kawai and Akira, 2010). RNA ligands stimulate splenocytes and HD11 cells that cause activation of IL-1β, IL-6, IL-8, and cytokines. This stimulation of cytokines is important for endosomal acidification due to chloroquine sensitivity (Kogut et al., 2005; He et al., 2006). Another research found contradictory results for TLR7 agonist loxoribine that downregulated pro-inflammatory cytokines (Kogut et al., 2005) and it was a contradictory observation reported for neutrophils in humans (Hayashi et al., 2003). A research report described that infection of avian influenza in chicken macrophages exhibited significant upregulation of TLR7 gene expression initially (Kawai and Akira, 2010), but the gene expression decreased later on.

## Expression Patterns of Tlrs During Different Avian Diseases

The expression of TLRs mRNA is not limited to the tissues involved in the immune system including spleen, thymus, tonsils, lymphatic vessels, and lymph nodes, but it is also found in the peripheral blood leukocytes, vital organs, pancreas, colon, small intestine, ovary, placenta, testis, prostate, and skeletal muscles (Zarember and Godowski, 2002). Moreover, the gene expression of TLRs has also been observed in various cells of the immune system including macrophages, NK cells, DCs, circulating leukocytes, adaptive immune cells, and non-immune cells like epithelial and endothelial cells and fibroblasts (Delneste et al., 2007). All the 10 TLRs are found on chicken heterophils and can be functionally activated *in vitro* with either TLR agonists or intact bacterial cells (Kogut et al., 2005, 2006, 2008). A broader and enriched expression pattern of TLRs has been observed in heterophils which suggests that heterophils might play a major role as first-line effector cells through TLR-induced signaling pathway (Kogut et al., 2005, 2006). In addition, TLRs are bridging molecules since they are also expressed on DCs that join innate and adaptive immunity (Gao et al., 2017). This is the reason that the induction of innate immunity by TLRs further stimulates adaptive immunity (Akira and Takeda, 2004). TLRs recognize the pathogen and convey the signal to APCs which kill the microbes by phagocytosis (Moresco et al., 2011; Sharma et al., 2013). Moreover, the presence of a total group of TLRs on immature DCs assists in their maturation process (Re and Strominger, 2011). The DCs initiate the immune response in the chicken gut by the production of cytokines and stimulate other immune competent cells. Different feed additives like phytochemicals have shown the potential to mediate the functions of DCs through both direct and indirect ways to effectively modulate the immune response (Teng and Kim, 2018).

The dynamic expression of TLRs may indicate the chances of genetic modulation and production of enhanced immunological response due to higher expression levels of TLRs in chicken. The TLR2 and TLR4 are only expressed on the surface of DCs, natural killer (NK) cells, and monocytes, but intracellular expression has also been noticed in the endothelial cells (Tamiru et al., 2019). Similarly, NK cells use a variety of cellular receptors, such as Ly49 and CD94 to induce immunological responses (Boudreau and Hsu, 2018). The DCs are grouped into CD11c positive myeloid and CD11c negative plasmacytoid (PDCs). These PDCs secrete IL-6, IL-10, IL-12, IP10, and tumor necrosis factor (TNF)-α as soon as they are exposed to pathogens and also regulate the TLR7 and TL9 dependent immune pathways (Katherine et al., 2020). The expression profiles of TLR3 and TLR7 have been found higher in lungs of the post-hatch chicks, while the expression of TLR7 increases with the age of the birds (Karpala et al., 2012). Different expression patterns of TLRs generate responses against pathogens of various poultry diseases; however, the individual role of TLRs in causing disease resistance is limited. The expression patterns of TLRs in a few infectious disease of poultry have been discussed below.

### Marek’s Disease

A comparative study between resistant and susceptible chicken embryo fibroblasts elucidated that resistant chicken shows higher TLR3, IL8, and TLR7 gene expression when treated with Marek’s disease and TLR3 ligand Poly(I:C; Haunshi and Cheng, 2014). The synthetic analogy to dsRNA like Poly (I:C) stimulates chTLR3 in DF-1 cells and spleen (Karpala et al., 2008) that acts as an adjuvant against Marek’s disease (Parvizi et al., 2012). The findings of Haunshi and Cheng (2014) are similar to the fact that the expression of chTLR3 increases in the bursa (Jie et al., 2013) and in lungs (Abdul-Careem et al., 2009) upon infection with Marek’s disease. The higher expression level of chTLR3 highlights the role in resistant birds in comparison with susceptible ones (Haunshi and Cheng, 2014). When CEFs were treated with TLR3 ligand, Poly (I:C) increased the expression level of IL-6, IL-1β, and IL-18 that subsequently resulted in 81% reduction in Marek’s disease (Zou et al., 2017; Bavananthasivam et al., 2018). The expression pattern of TLR in CEFs is different Bavananthasivam et al. (2018) as compared to Haunshi and Cheng (2014) study. This difference in expression confers that the genetic makeup of chicken may influence the TLR expression and develops resistance against diseases.

### Newcastle Disease

In Newcastle disease, a similar expression level of TLR3 and TLR7 is reported in chicken embryo fibroblasts and duck embryonic fibroblasts that activate host innate immune responses upon signaling cues received by pro-inflammatory cytokines and IFNs. The gene expression levels of TLR3 and TLR7 were higher in chicken embryo fibroblast due to the species differences in chicken and ducks (Kang et al., 2016). Additionally, the antiviral effect of TLR7 against Newcastle disease infection was also characterized in layers and reported presence of different haplotypes that responded to viral attacks as a front-line immune response (Rowland et al., 2018). In chicken bone marrow macrophage cell line HD11, TLR7 inhibited the replication of Newcastle disease (Zhang et al., 2018).

### Avian Influenza

The chTLR7 agonist Poly-C has shown to inhibit influenza virus replication in the chicken macrophage efficiently than TLR3 ligand Poly (I:C), but TLR3 also exhibited significant effect (Karpala et al., 2008; Stewart et al., 2012). The TLR3 agonist enhances the stimulation of IL-6, IL-12, and IFN-γ when these are used as an adjuvant with avian influenza virus (Liang et al., 2013). Moreover, the significant antiviral response of ligands of TLR2, 3, 4, 7, and 21, i.e., Poly (I:C), Pam3CSK4, LPS, and CpG, has been observed against H4N6 avian influenza virus infection. Ligands Pam3CSK4, CpG, and LPS reduce the growth of avian influenza virus in macrophages of chicken and increase expression profile of interferon regulatory factor-7, IL-1b, IFN-c, and IFN-b (Barjesteh et al., 2014). All TLR ligands reduce the shedding of virus with the greatest avian influenza virus immunity when treated with Poly (I:C; Paul et al., 2012). On the basis of these findings, it could be concluded that TLRs might have substantial ability to serve as an antiviral agent in chicken to control viral infections.

### Association of TLRs With Resistance/Susceptibility of Bacterial Diseases in Chicken

#### Salmonellosis

The TLR4, TLR15, TLR21, MD-2, ILs, IFNs, and iNOS have been reported as resistant genes against salmonella infections (Tohidi et al., 2012; Gupta et al., 2014). ChTLR21 acts as a receptor for microbial genetic material (Tamiru et al., 2019). MD2 is a specialized molecule needed by TLR4 to recognize LPS ligands (Shimazu et al., 1999). Upon salmonella infection, LPS serves as an inflammatory agent that is stimulated by TLR4. After inflammation, LPS binds to CD14 present on macrophage and these macrophages send signals to the TLR4/MD2 complex (Akashi et al., 2001). The said pathway takes part in the transcription of genes related to immunity during salmonella infection. MD2 is required for the LPS recognition of TLR4, intracellular distribution, and cell surface expression (Nagai et al., 2002). A number of TLR4 receptors vary among different chicken species, therefore, the expression levels of LPS-binding receptors vary between them (Dil and Qureshi, 2002). Two research studies proved that the susceptibility of salmonellosis is associated with chTLR4 (Beaumont et al., 2003; Leveque et al., 2003).

The relationship between the chTLR4 gene and susceptibility/resistance to salmonella infection has been studied (Leveque et al., 2003). Maximum resistant level of 93% has been observed in chicken due to TLR4 expression profiles and W1 (resistant) gene at NRAMP1 in comparison with those that have TLR4 and C alleles at locus NRAMP1 (58%). Furthermore, gene expression of TLR4, TLR5, and TLR21 increases substantially in *Salmonella enterica serovar Typhimurium* infected chicken, as soon as they bind with their respective ligands. Genetic expression of these TLRs suggests a positive role in resistance or susceptibility against *Salmonella serovars* (Shaughnessy et al., 2009).

#### Necrotic Enteritis

Gene expression of different TLRs in birds challenged with *C. perfringens* has been studied. It was observed that the infection upregulated the genetic expression of TLRs (TLR2 family, TLR15, and TLR21) and related genes that induced the TLR signaling in ilea and spleens (Lu et al., 2009). TLRs are the key activator of TNF-α production and also modulate the gene expression of TNF-α inducing factor homolog to activate NF-κB that turns out to be a stimulant for inflammation. In chicken exposed to Eimeria and Salmonella species, the assimilation of TNF-α modulation increases with the corresponding increase in the inflammatory cytokines (Higuchi et al., 2008). Inflammation and stimulation of innate response have been studied in necrotic enteritis (Lu et al., 2009), whereas the homolog expression of TNF-α inducing factor may have the ability to initiate the TNF-α response against *C. perfringens* in chicken. These findings highlight the importance of specific TLRs in birds’ disease resistance and further studies are required to explore the possible mechanism of action and related molecular targets.

## The Potential Regulation of Tlrs By Phytonutrients

Restricted uses of growth-promoting antibiotics motivate the use of alternate natural compounds for profitable chicken production. Natural plant-derived compounds are called phytonutrients that carry multiple healthy effects and also restrict the multiplication of microbial growth. Phytochemicals can promote quality food production in the chicken industry while keeping the taste of the food intact. Phytonutrients include natural plant extracts, essential oils, and phytochemical compounds that modulate innate/non-specific and humoral/specific immune responses in chickens (Karásková et al., 2015; Singh et al., 2016; Catanzaro et al., 2018). TLRs have been identified in B cells, macrophages, and heterophils, where host responses are mediated by enhanced cellular activity and cytokines production. In response to pathogen invasion and infection, TLRs elicit reactive oxygen species, inflammatory cytokines, upregulate inflammatory reaction, and promote host adaptive immune response (Keestra et al., 2013; Huang, 2017). No doubt, TLRs are essentially important in the innate immune system and play crucial roles in the host defence against microbial invasions, but overstimulation of TLRs disturbs the body homeostasis resulting in the production of excessive pro-inflammatory cytokines that subsequently leads to the onset of many autoimmune and inflammatory diseases. Thus, inhibition of TLRs signaling pathways has been considered as a potential therapeutic strategy to mitigate undesirable, disease-related inflammatory cascades (Gao et al., 2017: El-Zayat et al., 2019b).

Target-specific inhibition of TLRs can be sought through two possible ways (1) by blocking the binding of TLRs ligands to the corresponding receptor and (2) by interfering with the intracellular signaling pathways to stop the signal transduction. Based on these facts, it has been suggested that therapeutic interventions among TLRs pathways offer potential remedies to reverse chronic liver diseases (Catanzaro et al., 2018; Katherine et al., 2020). Many inhibitory agents for TLRs signaling have been developed to control excessive inflammation, such as small inhibitory molecules (synthetic or naturally derived chemical weak bases, e.g., antimalarial drugs), antibodies, oligonucleotides, lipid-A analogs, microRNAs, and new emerging nano-inhibitors (Gao et al., 2017). In addition, phytochemicals like curcumin and its analogs have shown to modulate JK/KB signaling pathway that results in NF-κB activation by inhibiting IκB phosphorylation and degradation (Ullah et al., 2020).

Moreover, 6-shogaol is a compound present in ginger which can inhibit the TRIF (Toll/interleukin-1 (IL-1) receptor domain-containing adapter inducing interferon)-dependent signaling pathway of TLRs by targeting TANK-binding kinase1, and, hence effectively modulate TLR-derived immune/inflammatory target gene expression induced by microbial infection (Park et al., 2009b).

Phytonutrients restrict the proliferation of T cells induced by PHA and also reduce the production of IL-2, nitric oxide (NO), LPS-dependent NF-κB-mediated inflammatory pathway, and augments NK cell cytotoxicity. Besides, these compounds also inhibit NF-κB, and MAPK signaling pathways to inhibit stimulatory signals necessary for T cell activation. Phytonutrients also impair the growth of pro-inflammatory (IL-12) cytokines. In several *in vivo* and *in vitro* studies, curcumin produces the preceding advantages of phytonutrients (Catanzaro et al., 2018). It is imperative to modulate the gene expression of TLR and/or LPS oligomerization of TLRs to prevent excessive energy loss and host cell damage. Several phytochemicals modulate the gene expression of TLRs or prevent oligomerization of TLRs by pathogen LPS. This ability of phytonutrients mediates the expression patterns of TLRs and opens the horizon for potential nutrigenomic interventions to modulate the immunogenic response in chicken. Effects of potent phytochemicals with superior ability to affect the regulation of TLRs are described as under:

### Thymol and Carvacrol

Inflammatory responses stimulated by TLRs *via* MyD88 pathways produce pro-inflammatory cytokines (Netea et al., 2004). Mammalian TLR4 can identify LPS which is a unique feature of Gram-negative bacteria, while TLR2 recognizes peptidoglycans of Gram-positive bacteria (Pasare and Medzhitov, 2005). During *C. perfringens* infections in broilers, mRNA expression of TLR2 was increased. On the other hand, supplementation of essential oils (EO) containing thymol and carvacrol decreased the mRNA expression of the same protein and improved resistance against the pathogens (Du et al., 2016). Thymol and carvacrol improve not only cellular immunity, but also increase the function of humoral system, whereas these chemicals also increase the expression of genes involved in chicken’s immune response (Awaad et al., 2014). Thymol and carvacrol inhibited inflammatory cell recruitment, pro-inflammatory cytokines, and oxidative impairment (Riella et al., 2012). However, pro-inflammatory response *via* cytokine production might damage the gut health and increase energy consumption (Lee et al., 2013a). Therefore, EO supplementation decreases TLR2 and pro-inflammatory cytokine that improves the health status of the gut (Du et al., 2016). When broiler birds were supplemented with carvacrol, TLR4 expression decreased considerably and inhibited the secretion of inflammatory cytokines (Liu et al., 2019). The blends of EO (25% thymol and 25% carvacrol) against necrotic enteritis have been well studied in broilers that were treated with *C. perfringens*. The supplementation of EO downregulated the TLR2 expression in challenged birds (Yin et al., 2017). This suggests that thymol and carvacrol regulate gene expression of TLR2 and TLR4 in diseased birds. Phytogenic feed additives including thymol and carvacrol have shown to downregulate the gene expression of TLR2 in chicken (Paraskeuas and Mountzouris, 2019).

### Andrographis Paniculata and Morinda Citrifolia

Andrographolide is a constituent of *Andrographis paniculata*, a medicinal plant that has been traditionally used to treat infectious diseases, inflammation, fever, and cold (Chandrasekaran et al., 2011). At present, andrographolide serves as a modulator of adaptive immune response and also regulates the TLRs activities (Thakur et al., 2014). Morinda citrifolia is commonly known as Noni which is a popular medicinal plant that contains multiple phytochemicals, such as gums and mucilages, carbohydrates, proteins, fats, amino acids, anthraquinone glycosides, flavonoids, coumarin glycosides, alkaloids, phenolic compounds, citric acid, and tannins (Nayak et al., 2011).

Kalmegh and Noni supplementation influenced the gene expression profile of TLR2, 3, 4, 5, 15, and 21 (Sunder et al., 2016). Phytochemicals and polysaccharides present in Noni fruit also modulated the NF-B signal transduction pathways in a dose-dependent manner (Desai et al., 2009). The upregulation of genetic expression of TLR3, 4, and 5 might be attributed to enhanced TLR signaling mediated by quercetin treatment which is a phytochemical found abundantly in Noni and accelerates IFN-γ production (Park et al., 2009a). These antiviral products upregulate TLR3, TLR4, and TLR5 genes (Tanabe et al., 2003). The higher gene expression of TLR3, TLR4, and TLR5 and lower gene expression of TLR7 indicate the antiviral and antibacterial activities of IFN-γ. Andrographolide induces the APK and PI3K signaling pathways that activate macrophages (Wang et al., 2010). Moreover, andrographolide is more effective in downregulate gene expression of TLR7 and TLR8 which suggests that the modulation of cytokine might be due to the inhibition of TLR7 and TLR8 gene expression (Thakur et al., 2014). TLR7 and TLR8 are similarly expressed in HL-60 cell lines treated with andrographolide and revealed the anti-inflammatory effect of andrographolide (Thakur et al., 2015). It also increases cellular apoptosis and downregulates NF-κB protein and TLR4 expression to control the inflammatory response (Gao and Wang, 2016). The significant effect of andrographolide to downregulate expression profiles of TLR3, TLR7, and TLR8 in the intestine of *Monopterus albus* suggested its key role as health promotor in aquaculture (Shi et al., 2020). However, more detailed dose–response studies are necessary to reaffirm this assertion (Thakur et al., 2014).

### *Allium sativum* and *Ocimum sanctum*

*Allium sativum L*. is commonly known as garlic and it has been extensively used in various medical treatments for a long time. It has an immunomodulatory effect that increases T-lymphocyte blasto-genesis, phagocytosis, and cytokine (Hodge et al., 2002). S-Allylcysteine is a key compound in garlic extract that mediates repressive effects on NF-κB as it also serves as a transcriptional modulator during an adaptive immune response and stands as a sole regulator of proinflammatory gene expression (Ho et al., 2001). Allicin is also an antibiotic constituent of garlic that suppresses the bacterial growth in intestines and also inhibits fungal growth that produces aflatoxin (Ho et al., 2001). Holy basil (*Ocimum sanctum*) has the features of anti-inflammation, wound-healing, and modulates humoral immunity (Cohen, 2014). EO and biologically active compounds (ethanol, methanol, linalool, and eugenol) of *O. sanctum* have resourceful antibacterial functions especially against *Shigella* spp.*, Staphylococcus aureus, Salmonella typhi, Bacillus pumilus, E. coli*, and *Pseudomonas aeruginosa* (Prakash and Gupta, 2005). Monoterpene, a component of *O. sanctum*, is a phenolic compound in nature and exhibits immunomodulatory effect by increasing IFN-γ, IL-4, T-helper, and NK cells that result in phagocytic activity (Mondal et al., 2011). In a study, *Eimeria acervuline* challenged chickens were fed garlic metabolites (propyl thiosulfinate and propyl thiosulfinate oxide) and these metabolites significantly decreased mRNA expression of TLR3 and TLR5 and also increased anti-inflammatory response (Kim et al., 2013a). Garlic powder and leaf powder of Holi basil promote mRNA translation of TLR2, TLR4, and TLR7 of broilers (Sheoran et al., 2017). Since TLR2 and TLR4 increase innate immunity against various Gram-positive and Gram-negative bacteria, therefore, changes in their genetic expression through garlic powder and leaf powder of Holi basil suggest a positive effect against bacterial infections. TLR7 recognizes viral nucleic acid and increases the level of TLR7 either by garlic powder alone or in combination with leaf powder. Allicin and Ajoene are present in garlic that enhance the host innate immunity against plasmodium and HIV infection, respectively (Feng et al., 2012).

### Curcumin and Other Dietary Flavonoids

Turmeric is the most extensively studied medicinal plant among the Curcuma genus and possesses many phytochemicals to trigger immunity (Li et al., 2011). Curcumin (diferuloylmethane) has properties of antioxidant, anti-inflammation, lipid peroxidase inhibitor, antiviral, free radical scavenger, antimicrobial, antitumor, antiprotozoal, and immunity enhancer (Pulido-Moran et al., 2016; Amalraj et al., 2017). Curcuminoids (a mixture of curcumin, dimethoxy curcumin, and bisdemethoxycurcumin) are effective anti-inflammatory agents by acting through multiple mechanisms, such as suppression of NF-κB, inhibition of cyclooxygenase-2, downregulation of the metastatic gene products, cell proliferation, and anti-apoptotic (Aggarwal et al., 2006). Curcuminoids also modulate the proliferation and cellular response of macrophages, B cells, neutrophils, T cells, DCs, and natural killer (NK) cells (Chandrasekaran et al., 2013). LPS activates TLR4 that stimulates MAPK and NF-κB pathways to produce cytokines (Kagan and Medzhitov, 2006). For that reason, reduction of the inflammatory response is needed *via* downregulation of NF-κB pathway. Supplementation of curcumin downregulates the gene expression of TLR4, and associated downstream molecules (NF-κB, MyD88, TNF-α, 1l-1β, and IL-6) in laying hens (Nawab et al., 2019). TLR4 activates MyD88 and regulates NF-κB activation (Karnati et al., 2015). Moreover, IL1β, IL6, IL12, IL18, and TNF15 increase in chicken macrophages treated *in vitro* with organic extract of turmeric (Lee et al., 2010). Turmeric also downregulates CD28, myeloperoxidase, and lactotransferrin in broiler birds that are associated with inflammatory response (Kim et al., 2013b).

A study reported the effect of 10 ethanolic extracts including Castanea sativa leaves, barks of Cinchona pubescens, Cinnamomum verum, Salix alba, Rheum palmatum root extract, Alchemilla vulgaris plant, Humulus lupulus cones, Vaccinium myrtillus berries, Curcuma longa root, and Arctostaphylos uvaursi leaves which worked as an anti-inflammatory drug. These extracts significantly mitigated TLR4 and TLR2 signaling pathways (Schink et al., 2018). Resveratrol acts as an anti-inflammatory agent that is an important phytoalexin found in various fruits like berries, peanuts, and grape skins (Saleh et al., 2021). It inhibits TLR4 expression in macrophages and heart tissues and reduces inflammation (Li et al., 2015; Tong et al., 2020).

A group of flavonoids including flavones, flavonols, isoflavones, flavanones, anthocyanidin, and flavanols demonstrated anti-inflammatory properties. Flavonoids are polyphenols that serve as antioxidant and anti-inflammatory agents in diet and regulate TLR gene expression (Pérez-Cano et al., 2014). Significant downregulation of TLR4 has been noticed after the treatment with flavonoids (Pérez-Cano et al., 2014). Flavonoids have shown to mediate the molecular targets and TLR-mediated signaling pathways. The potential effects of flavonoids on immunoregulatory response in the body are mediated through three primary levels (1) by modulating the composition of the microbiota (2) by mediating the expression and activation of TLRs, and (3) through modulating the downstream signaling pathways involved. The combined action of all these pathways might explain the crucial utility of flavonoids in preventing various diseases and immune-related disorders in different avian species (Pérez-Cano et al., 2014). The suppression of TLRs activation by flavonoids offers the opportunity to develop new and alternative therapeutic interventions for the treatment of inflammatory diseases.

### Effect of Yeast-Derived Products

Yeast (*Saccharomyces cerevisiae*)-based probiotic has been a promising substitute for antibiotics, as it is capable to modulate bacterial population (Trckova et al., 2014). Yeast can stimulate an immune response by providing binding sites to pathogens (Gao et al., 2008).

The immune modulation mechanism in yeast has not been fully understood. Polysaccharide is the main constituent of the yeast cell wall (YCW) that partially consists of β1, 3–1, 6-glucans, and mannan which modulate PRRs expression to enhance the innate immunity (Mogensen, 2009). Recognition of YCW by PRRs may activate macrophages and DCs of innate immunity following the modulation of cytokines (IL-4 and IL-10) that facilitate antibiotic production (Haghighi et al., 2006).

Gene expression of chTLR2b and chTLR4 supplemented with yeast-derived macromolecules improves the spleen and bursa of Fabricius (Yitbarek et al., 2013). Dietary nucleotides increase cell-mediated immunity, improve host resistance, and humoral immunity against invaded bacteria (Hess and Greenberg, 2012). Higher TLR2b level enhances barriers of the gastro-intestinal epithelium to block the invading pathogens (Chen et al., 2007).

Mannan oligosaccharides (MOS) are yeast-derived carbohydrates that have great potential to control necrotic enteritis in chicken, while simultaneously control pathogenic invasion (Hajati and Rezaei, 2010). ChTLR2 acts as a receptor for lipoproteins and LPS (Fukui et al., 2001). Therefore, broiler chicken fed on MOS challenged with C. perfringens show upregulated TLR2b gene expression (Yitbarek et al., 2012). However, zymosan derived from Saccharomyces cerevisiae is recognized through TLR2 which initiates the cascade of pro-inflammatory stimulation (Sato et al., 2003). Thus, the inclusion of MOS in broiler chickens might benefit to perform the proper functions of innate immunity in the ileum. Upregulated TLR4 gene expression in caecal tonsil and ileum in MOSC-treated birds prove that TLR4 is a key receptor to identify the LPS (Yitbarek et al., 2012). Studies implicate that upregulated gene expression of TLR4 is associated with resistance to Salmonella Saccharomyces cerevisiae, glucuronoxylomannan, and Candida albicans derivatives in chicken (Chaussé et al., 2011).

Various components of YCW produce immunomodulatory functions, and supplementation of even 0.05% YCW enhances cell-mediated and humoral immune reactions in chicken (Gómez-Verduzco et al., 2009). Particularly, β-glucans supplementation stimulates innate immunity and increases resistance to *S. enterica* (Lowry et al., 2005). Nucleotides of yeast and YCW have also immunomodulatory properties (Hess and Greenberg, 2012). Genetic expression of TLR4 gene was upregulated in chicken supplemented with nucleotide-rich diet (Alizadeh et al., 2016) and YCW supplementation also upregulated TLR2b gene expression (Alizadeh et al., 2016). This may be due to a noteworthy level of mannan and β1,3–1,6-glucan (Sato et al., 2003). Mannan oligosaccharides improve chicken production and increase innate immunity. Its use in the diet may also exert beneficial effects. This discussion highlights the importance of yeast and yeast-derived products during gene regulation of TLRs.

A study was conducted to evaluate the effect of probiotics (*Lactobacillus acidophilus*) on different genes expression levels of TLRs in chicken’s cecal tonsil and the TLR2, TLR4, and TLR5 gene expression were different in chickens fed with probiotic mixed diet in comparison with the birds in the control group (Asgari et al., 2018). Same probiotic supplementation increased the TLR2 regulation in cecal tonsils of *S. enteritidis*-infected chickens that helped in lowering the infection level. The previous studies showed that *Lactobacillus*-based probiotics could reduce the level of pro-inflammatory cytokines in the intestine of *S. Enteritidis*-infected chickens and increased the gene expression of TLR2 in their cecal tonsils (Penha Filho et al., 2015). The effects of probiotics on genetic expression of TLRs in dairy cow were also evaluated, and gene expression of TLR2, TLR6, TLR7, and TLR8 was downregulated. Studies in cattle TLRs suggested that probiotic behaved like anti-inflammatory agents and control TLRs genetic expression and innate immune response (Adjei-Fremah et al., 2018).

### Effect of Micronutrients

Inflammatory responses of TLR2 and TLR4 have also been studied against high-fat diet in rats (Wan et al., 2014; Lee et al., 2015). These treatments downregulated the gene expression of TLR2 and TLR4 in CD14 monocytes and impaired their functions (Wan et al., 2014). This diet activated the macrophages that significantly increased stimulation of NF-κB and IL-6 (Lee et al., 2015). TLR-dependent vitamin D mediated innate immunity has also been studied (Arababadi et al., 2018). Innate immunity regulation by vitamin D was also associated with TLRs regulation (Sadeghi et al., 2006). It was confirmed that TLR4 caused the activation of APCs that reduced the inflammatory response of innate immunity (Gambhir et al., 2012; Calton et al., 2015).

## Polymorphism in Different Tlrs in Chicken

Polymorphic variants in TLRs may control the reaction of hosts against different pathogenic microbes, and these phenomena control the susceptibility and resistance against diseases (O’Dwyer et al., 2013). These variations may also affect the recognition patterns of ligands by TLRs to differentiate host resistance to pathogenic infections. The single-nucleotide polymorphisms (SNPs) found within the PRRs change the structural orientation of the receptors and associated interactive features between the ligands and their corresponding receptors (Skevaki and Pararas, 2015). These topological variations influence the signaling pathways and also enable to recognize different pathogens.

A study reported 27 polymorphic sites in the amino acid sequence of chTLR1; 14 in type 1, while 13 in type 2 (Ruan and Zheng, 2011). Scientists declared that variations in the sequence of amino acids G560S and T130A present in LRR site of type 1 and C228S, F129L, G285S, and L275P in type 2 LRR site might change PAMP recognition by these TLRs. LRRs are the extracellular domains of TLRs to facilitate functional domains for the recognition of ligands (Jin and Lee, 2008). Moreover, two distinctive polymorphic sites have been found; first (A645T) in chTLR1 type 1 and second (L275P) in chTLR1 type 2, and both occur in LRR domains in White-Leghorn chicken (Table 2). These variants characterize resistance against salmonellosis (Wigley, 2004).

**Table 2:** Allelic variation and major single-nucleotide polymorphisms in the different TLRs in chicken.

Gene	Polymorphism	Breed	Reference
TLR1 Type 1	T130A	RY	Ruan and Zheng, 2011
	G560S	WS	
	A645T	WL	
	I388L	NB	Haunshi et al., 2018
TLR1 Type 2	F129L	BF	Ruan and Zheng, 2011
	C228S	NN3, LB	
	L275P	WL	
	G285S	WS	
TLR2 Type 1	Q45R	BF	Ruan et al., 2012a
	L115P	NN3	
	H232Y	NN3	
	E284G	BW	
	T494A	NN3	
TLR2 Type 2	A22V	BW	Ruan et al., 2012a
	V66L	BF	
	I311V	HL	
TLR3	D14V	HL, WS	Ruan et al., 2015
	R345S	BW, HL, NN3, WL	
	G362E	BF, BW, HL, LB, LH, NN3, RY, WL, WS	
	R459K	BF, BW, HL, LH, NN3, RY, WL	
	A540V	LB, WS	
	A649V	BW, HL, LH, NN3, RY, WL, WS	
	D68V	NB, GH	Haunshi et al., 2018
	K92Q	NB, GH	
	N98S	NB	
	H316Q	NB	
	L317K	NB	
	R399S	WLH, NB, GH	
	R513K	WLH, NB, GH	
TLR4	K83E	NN3, HL, LB, LH, BB, BY, CH, HG, LY, TH, LS, WE, WL, XJ	Ruan et al., 2012b; Li et al., 2017
	R261K	LB	Ruan et al., 2012b
	G225E	WL	Leveque et al., 2003
	D301E	NN3, HL, LH	Leveque et al., 2003; Ruan et al., 2012b; Li et al., 2017
	R343K	NN3, HL, LH, BF, BW, BB, BY, CH, HG, LY, TH, LS, WE, WL, XJ	
	Y383H	WL, BB, BY, CH, HG, LY, TH, LS, WE, WL, XJ	Ruan et al., 2012b; Li et al., 2017
	F427V	RY	Ruan et al., 2012b
	P551T	LB, RY	Leveque et al., 2003
	E574D	RY	
	R611Q	NN3, BB, BY, CH, HG, LY, TH, LS, WE, WL, XJ	Leveque et al., 2003; Ruan et al., 2012b; Li et al., 2017
TLR7	V91L	RY, WS	Ruan et al., 2015
	S135T	WS	
	P669S	HL, RY, WL, WS	

*BF, Beijing Fatty; BW, Beijing White 939; HL, Hy-Line Brown; LB, Laiwu Black; LB, Luhua; NN3, Nongda no. 3; RY, Royal; WL, White Leghorn; WS, White-Feather Silky; GH, Ghagus; NB, Nicobari; BB, Big Bone; BY, Beijing-you; CH, Chahua; HG, Henan Game; LS, Langshan; LY, Liyang; TH, Taihe; WE, White Earlobes; and XJ, Xianju*.

Similarly, 10 polymorphic sites in the amino acid sequences of TLR2 type1 and type 2 have been reported among seven chicken breeds, i.e., six and four in type 1 and type 2, respectively (Ruan et al., 2012a). Five SNPs have been found in LRR of TLR2 type 1 (Q45R, L115P, H232Y, E284G, and T494A) while three were LRR of type 2 (V66L, I311V, and A22V). However, TIR domain is similar to the IL-1 receptor that is highly conserved and interacts with adapter proteins, such as MyD88 (Verstak et al., 2009). Various polymorphic sites also report in the TIR domain (Table 2) that cause loss of MyD88 binding and reduce TLR2/TLR4 signaling, because TIR is the functional domain of subsequent recruitment of intracellular adapter proteins (Nagpal et al., 2009; Werling et al., 2009).

A study reported nine polymorphisms in the amino acid chain of chTLR4 including eight in extracellular sites, while only one in cytoplasmic sites (Ruan et al., 2012b). Out of these, nine reported SNPs, five were like the SNPs in the LRR region as shown in Table 2 (Leveque et al., 2003). Similarly, nineteen amino acid variants represented 10 novel mutations, while nine were reported previously. Seven new and four old mutations (E83K, R343K, H383Y, and Q611R) have been featured in LRR (Li et al., 2017). Moreover, E83K of TLR4 has more resistance against SE, and E83K mutation has been documented as AA/AG genotypes because of G to A substitution at nucleotide 247, and GG is known as a wild genotype. However, this SNP shows significant resistance against salmonella with prominent results in the GG genotype (Li et al., 2017).

Similarly, genotype GG has been associated in Sentul chicken against salmonella and Newcastle disease (Mamutse et al., 2018). However, all genotypes (AA, AG, and GG) were also found with a similar resistance pattern against salmonella in Kampung chicken (Ulupi et al., 2013). A group finds polymorphism in TLR4 gene’s exon 2 in 14 Chinese chicken including Red Jungle fowl and Tibetan chicken. Particularly, these chickens are more resistant to disease, and they are only BB at exon 2 of TLR4, while the rest of all breeds is AA, BB, and AB. Here, BB has been reported as a wild type at TLR4 exon 2, and AA genotype is because of G/A mutations at nucleotide 114 and 142 of exon 2, respectively, as reported as G114A and G142A. The genotype BB may be beneficial for immunity in chickens, and the B allele might have a significant association with resistance (Liu et al., 2011). They also found two mutations in the amino acid sequence of TLR4, G114A, and G142A. Further investigation to find the relationship between these SNPs and resistibility to disease will reveal selective breeding programs in the future.

ChTLR3 and chTLR7 have the potential to increase innate immunity against viral attacks as discussed in this review. Therefore, various polymorphic regions might play frequent roles in resistance or susceptibility of disease in chickens. A study has published six SNPs (R345S, D14V, G362E, A540V, A649V, and R459K) in the LRR region of TLR3, while a single one (T713S) has been found in TIR domain. Three polymorphic sites have been found in the LRR region of TLR7 (V91L, S135T, and P669S), while one (V876M) has been located in the TIR domain as shown in Table 2 (Ruan et al., 2015). Ten SNPs in the LRR region of TLR3 have been observed in Indian local chicken breeds and eight in the LRR domain as shown in Table 2 (Haunshi et al., 2018). Polymorphic regions in TLR3 and TLR7 might exert potential effects on host responses against viruses to establish patterns of variable disease resistance or susceptibility (Al Qahtani et al., 2012; O’Dwyer et al., 2013). A number of reports have presented polymorphic regions in TLR3 gene that are associated with resistance against viral load (Kindberg et al., 2011; Sironi et al., 2012; Svensson et al., 2012; Lee et al., 2013b; Studzińska et al., 2017). A parallel study also reported significant relationship of different TLR3 variants in the susceptibility or resistance against various chicken diseases. These are non-synonymous changes in SNPs due to change in amino acid sequence. These can be beneficial, harmful, or neutral against proteins functions, while it may be neutral if SNPs have synonymous substitution (Downing et al., 2010).

The available variations in chTLRs suggest a positive selection of resistance or susceptibility against diseases. A sufficient research gap is yet to be exploited to identify the association of SNPs with resistance against microbial infections in chicken. Identification of associated polymorphic regions may contribute to the genomic selection of chicken and help to design future breeding programs. There is also a dire need to conduct some association studies among these variants and resistance in chicken for the improvement of commercial chicken.

## Conclusion

Ten different TLRs activate TLR signaling pathways including ChTLR1, 2, 3, 4, and 7 which elicit essential immunogenic and inflammatory responses during different viral and bacterial diseases. Several nutrients and phytochemicals have proven as an excellent source to trigger innate immunity *via* stimulation of TLRs in chicken suggesting that some TLRs are nutrient specific. Yeast derivatives (as probiotic and prebiotic) have also been revealed as potential modulators of TLRs to enhance the immune response in chicken under health and disease conditions. Such interventions suggest the nutrigenomic potential of TLRs to improve the health status and production through dietary supplementation in chicken feed with specific nutrients particularly phytonutrients. Moreover, variations in TLRs have been identified that link potential association with disease susceptibility and resistance in chicken. Such genetic variations including SNPs are particularly useful for the genomic selection of chicken to produce birds with better genetic resistance and resilience against different diseases. However, a lot of work is needed to explain the role of nutrients and phytochemicals that modulate the genetic expression of TLRs.

## Author Contributions

QL and HP: conceptualization and project administration. F-uH, SR, HP, and QL: resources. MR, SR, WY, WA, and F-uH: data searching. MR and SR: writing and original draft preparation. F-uH, SR, WY, WA, QL, and HP: review and editing. QL: funding acquisition. All authors read and approved the final version of the manuscript.

## Conflict of Interest

The authors declare that the research was conducted in the absence of any commercial or financial relationships that could be construed as a potential conflict of interest.

## Publisher’s Note

All claims expressed in this article are solely those of the authors and do not necessarily represent those of their affiliated organizations, or those of the publisher, the editors and the reviewers. Any product that may be evaluated in this article, or claim that may be made by its manufacturer, is not guaranteed or endorsed by the publisher.
